# Could misreporting of condom use explain the observed association between injectable hormonal contraceptives and HIV acquisition risk?^☆^^☆☆^^★^

**DOI:** 10.1016/j.contraception.2016.12.003

**Published:** 2017-04

**Authors:** Jennifer A. Smith, Renee Heffron, Ailsa R. Butler, Connie Celum, Jared M. Baeten, Timothy B. Hallett

**Affiliations:** a Department of Infectious Disease Epidemiology, Imperial College London, London, UK; b Department of Global Health, University of Washington, Seattle, USA; c Department of Epidemiology, University of Washington, Seattle, USA; d Department of Medicine, University of Washington, Seattle, USA

**Keywords:** DMPA, Injectable hormonal contraceptives, HIV, Condom use, Misreporting bias, Mathematical modelling

## Abstract

**Objective:**

Some observational studies have suggested an association between the use of hormonal contraceptives (HC) and HIV acquisition. One major concern is that differential misreporting of sexual behavior between HC users and nonusers may generate artificially inflated risk estimates.

**Study design:**

We developed an individual-based model that simulates the South African HIV serodiscordant couples analyzed for HC–HIV risk by Heffron et al. (2012). We varied the pattern of misreporting condom use between HC users and nonusers and reproduced the trial data under the assumption that HC use is not associated with HIV risk. The simulated data were analyzed using Cox proportional hazards models, adjusting for the reported level of condom use.

**Results:**

If HC users overreport condom use more than nonusers, an apparent excess risk could be observed even without any biological effect of HC on HIV acquisition. With 45% overreporting by HC users (i.e., 9 out of every 20 sex acts reported with condoms are actually unprotected) and accurate condom reporting by nonusers, a true null effect can be inflated to give an observed hazard ratio ($\hat{\mathrm{HR}}$) of 2.0. In a different population with lower overall reported condom use, artificially high $\hat{\mathrm{HR}}$s can only be generated if non-HC users underreport condom use.

**Conclusion:**

Differential condom misreporting can theoretically produce inflated $\hat{\mathrm{HR}}$ values for an association between HC and HIV even without a true association. However, to produce a doubling of HIV risk that is entirely spurious requires substantially different levels of misreporting among HC users and nonusers, which may be unrealistic.

**Implications:**

Considerably differential amounts of condom use misreporting by HC users and nonusers would be needed to produce entirely spurious observed levels of excess HIV acquisition risk among HC users when there is actually no true association.

## 1 Introduction

One of the most significant successes in global health has been the development of safe and effective methods of family planning and the expansion of their use in low- and middle-income countries [1]. A major challenge to global health in many countries, especially those in southern Africa, remains high HIV incidence in women of reproductive age [2]. It is therefore of substantial concern that some evidence suggests that the use of hormonal contraceptives (HC), particularly injectables, could increase women's risk of acquiring HIV infection, with recent meta-analyses suggesting a 1.4- to 1.5-fold increase in risk for women using the injectable depot medroxyprogesterone acetate (DMPA) [3], [4], [5], [6]. The totality of analyses from observational studies assessing the potential association has produced conflicting results that are difficult to reconcile [7], [8], [9], [10], [11], [12], [13], [14], [15], [16], [17], [18], [19]. A challenging issue for these studies has been to appropriately adjust for behavioral patterns in the groups of women exposed and unexposed to HC. In particular, differences in condom use, coital frequency and the self-reporting of these measures have been hypothesized to artificially elevate risk estimates and produce spurious observed associations between HC use and HIV acquisition. Additionally, reported levels of condom use have varied widely across HC users and non-HC users in the observational studies among different populations, and this could impact the magnitude of any effect, as there is more room for overreporting when reported condom use is high and vice versa.

Despite efforts to promote condom use in conjunction with the use of hormonal contraception, condoms may be used less frequently by HC users compared to nonusers, perhaps because they are not relied on as the primary strategy for pregnancy prevention [20], [21]. Considering this trend and all else being equal, the unadjusted HIV incidence rate among these women would be higher than others even in the absence of a biological effect of injectable hormonal contraception on HIV risk. In order to determine whether there is evidence of an additional risk of HIV infection among HC users, condom use must be accounted for in statistical analyses. All observational studies to date have collected data on condom use via self-report, which is difficult to collect reliably [22], [23]. Furthermore, the degree of social desirability and recall bias related to condom reporting could vary according to contraceptive choice.

One observational analysis found a statistically significant twofold increase in HIV acquisition risk for women using combined oral contraceptives (COC) or injectable HC (hazard ratio [HR]=1.98 [95% confidence interval (CI) 1.06–3.68]) [7]. Our objective was to use mathematical modeling to understand the patterns of misreported condom use that would be necessary to reproduce that risk estimate in the absence of a true biological risk.

## 2 Methods

### 2.1 Model structure and parameterization

We developed an individual-based simulation model that reproduces the behaviors of the subset of stable HIV serodiscordant heterosexual couples of the South African sites in the Partners in Prevention HSV/HIV Transmission Study that were analyzed in Heffron et al. [7] Partners HSV/HIV was a multinational prospective HIV prevention clinical trial among HIV serodiscordant couples in which the HIV-infected partner had CD4 >250 cells/mm^3^ and was not eligible for antiretroviral therapy (ART) by national guidelines at enrolment [24], [25]. The full model specification is described in [26]; in brief, the model tracks HIV transmission, disease progression and treatment and includes the composition of couples by sex, age, current CD4 cell counts, variations in coital frequency within stable partnerships, outside sexual partners and HC use (Table 1, Table 2). We capture differences between HC users and nonusers with respect to condom use only and assume that all other characteristics are equivalently distributed across groups.

### 2.2 Misreporting condom use

**Fig. 1:**
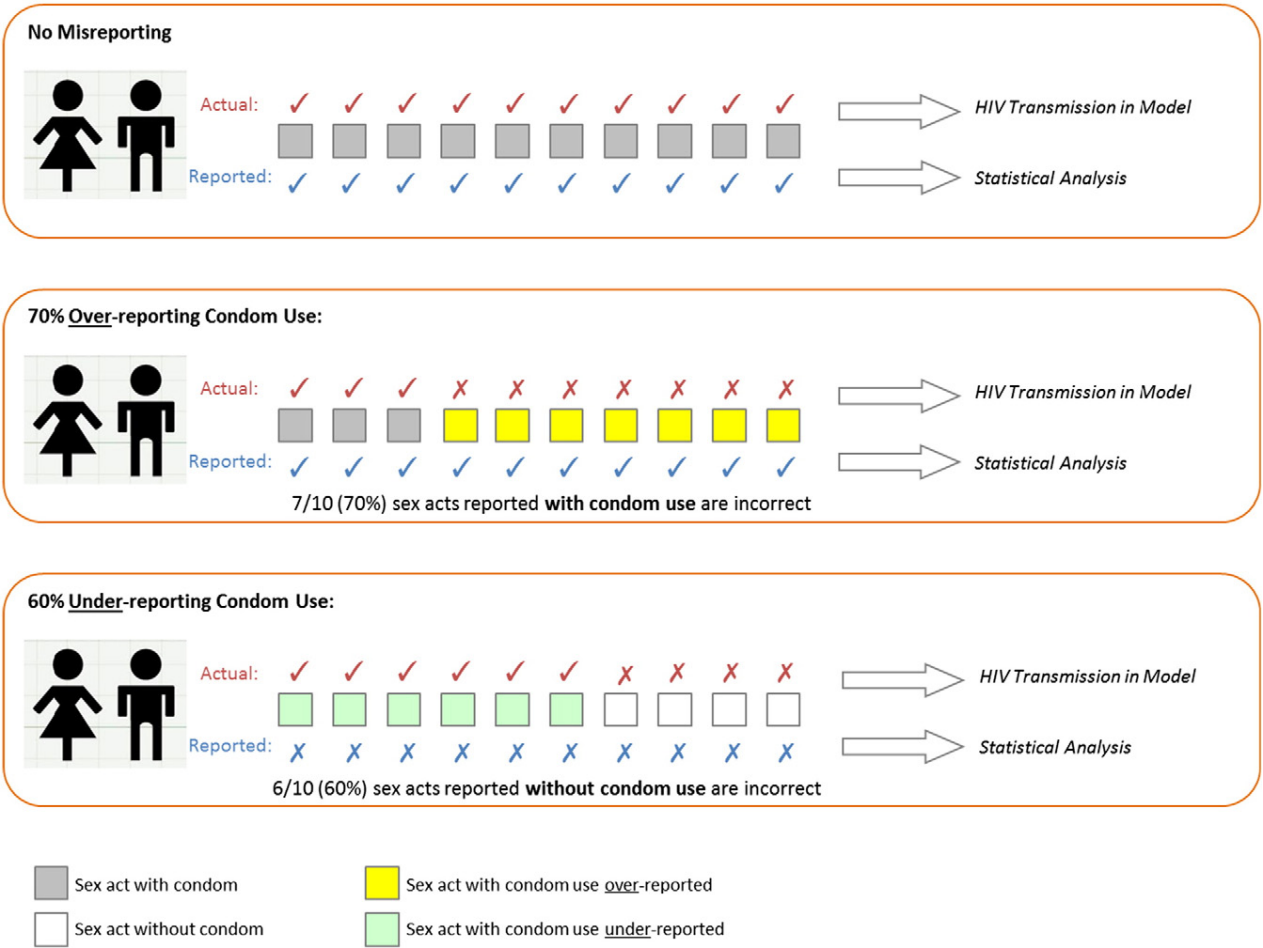
Schematic of condom use and condom reporting in the model. No misreporting means that all sex acts are reported accurately with respect to condom use. −100% means that all sex acts using condoms are reported as unprotected (underreporting); 100% means that all unprotected sex acts are reported with condom use (overreporting). In this example, with 70% overreporting, an individual who reported using condoms in 10 out of 10 sex acts would be assumed to actually use a condom in only 3 sex acts (70% of their reported sex acts with condom use are reassigned to unprotected sex acts). With 60% underreporting (or −60% misreporting), an individual who reported using condoms in 0 sex act out of 10 would be assumed to actually use a condom in 6 (60% of their unprotected sex acts are reassigned as using condoms).

The model records true and reported condom use, HC use and HIV acquisition to create a set of simulated data that is analogous to the epidemiological data, with oral (22% of HC users) and injectable (both DMPA and norethisterone enanthate, Net-En, together 78%) HC grouped together to replicate the primary analysis [7]. Crucially, the model distinguishes between the actual pattern of condom use (which affects HIV transmission in the model) and the pattern that is reported (used in the statistical analysis; Fig. 1). To simplify the model, we assume that there is no contraceptive switching through the 2-year follow-up period, with the exception of condom use, which may vary month to month. Condoms are assumed to reduce HIV transmission by 78% per sex act [27]. Each individual is simulated over the 2-year study.

Condom misreporting is modeled by manipulating the proportion of sex acts per month that are assumed to occur with and without condoms relative to the proportion of sex acts for which condom use is reported (Fig. 1). Independent misreporting parameters were set for HC users and non-HC users, varying between complete underreporting (labeled −100% misreporting) and complete overreporting (+100%). Accurate reporting is defined as 0% misreporting. Overreporting was implemented as nm=c.nc100, where *n*_m_ is the number of misreported sex acts per month, *n*_c_ is the number of sex acts with reported condom use and *c* is the misreporting parameter. Underreporting was implemented as nm=c.nu100, where *n*_m_ is the number of misreported sex acts, *n*_u_ is the number of reported unprotected sex acts and *c* is the misreporting parameter. For both, condom use in the misreported sex acts was then reassigned as appropriate.

### 2.3 Model scenarios

In the data, most couples reported either consistent condom use or none at all. The proportion that reported no unprotected sex was similar among HC users and nonusers (91.4 vs. 92.5%; p=0.4^7^). Therefore, in the model, we initially assigned the same proportion of couples in each group to always report using condoms at the start of each simulation, calculated to reproduce the observed proportion with no unprotected sex (91.1% consistent condom use, Scenario 1) and the null hypothesis of HR=1.0.

We then repeated the analysis in two additional scenarios with different assumptions (Table 2). In Scenario 2, we assumed the same level of reported condom use as Scenario 1 but added an underlying weak association between HC use and HIV acquisition risk (HR=1.2).

Serodiscordant couples in the Partners HSV/HIV Transmission Study reported a much lower level of unprotected sex than participants in many other observational studies of HC and HIV, including another study of serodiscordant couples [19]. Therefore, in Scenario 3, we investigated the impact of misreporting condom use under the assumption that 50% of couples report no unprotected sex and the null hypothesis of HR=1.0.

### 2.4 Statistical analysis

We varied the patterns of condom misreporting from 90% underreporting to 90% overreporting for HC users and nonusers separately in each model scenario. The model generates a simulated dataset that is analyzed in a Cox proportional hazards (PH) model, adjusting for any reported unprotected sex over the simulated study period. This gives an estimate of the $\hat{\mathrm{HR}}\;$for HIV acquisition risk for women due to HC use in the model (we designate the model-estimated HR as $\hat{\mathrm{HR}}$ throughout) that is analogous to the primary statistical methods of Heffron et al. Although the observational evidence for an association between HC use and HIV acquisition is strongest for DMPA [6], we group oral and injectable HC together in the statistical analysis for consistency with the primary analysis and because both methods are effective against unintended pregnancy and not expected to generate different degrees of misreporting. HC use in the model is assumed to be consistent throughout the study period.

The model and statistical analysis is run varying misreporting in HC users and nonusers independently from −90% to 90% in 5% increments, repeated 100 times at each combination. A smooth surface is then fit to the geometric means of the $\hat{\mathrm{HR}}$s at each grid point using locally weighted scatterplot smoothing with a quadratic polynomial (LOESS). Reported point estimates refer to the fitted surface, and the uncertainty bounds represent 90% of the variation in model $\hat{\mathrm{HR}}$s at that point (the 5th and 95th percentiles in the distribution of model $\hat{\mathrm{HR}}$s). All simulations and statistical analyses were performed using MATLAB and Statistics Toolbox Release 2012b [28].

## 3 Results

### 3.1 Scenario 1: reported condom use from the Partners in Prevention HSV/HIV transmission study

With the reported condom use and no misreporting, HIV incidence among the serodiscordant couples in the model is 3.9 per 100 person-years (py; 90% of model variability: 3.2–4.4) from linked and unlinked infections, similar to the 4.1 per 100 py observed in the epidemiological data.

**Fig. 2:**
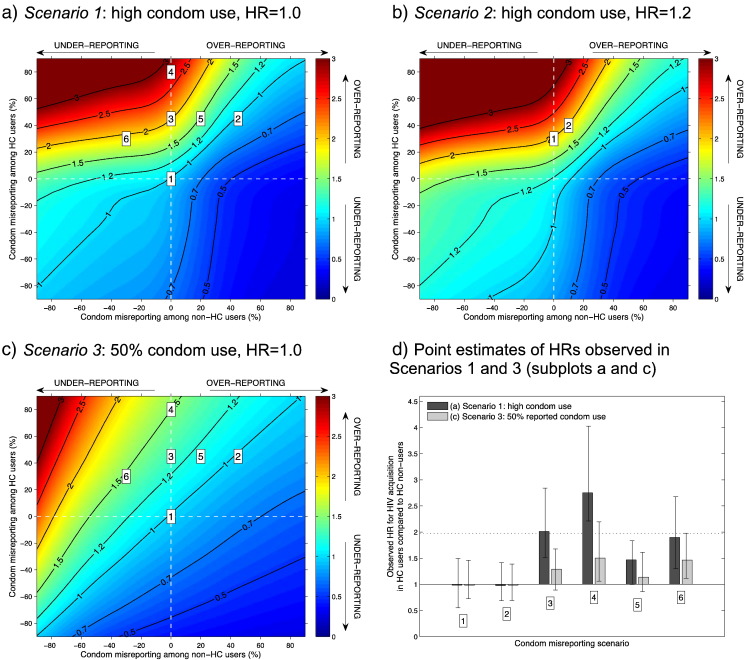
Model-generated HRs with different levels of reported condom use and underlying true HR. Point estimates of HRs observed in the simulated data under different levels of misreporting condom use among HC users (15% of women; vertical axis) and nonusers (85% of women; horizontal axis). (a) Scenario 1: reported condom use from the Partners in Prevention HSV/HIV Transmission Study, HR=1.0. Ninety-one percent of both HC users and nonusers report consistent condom use. Specific misreporting biases are labeled 1–6 which are discussed in the text and plotted in Fig. 2d (dark gray). These are (1) no condom misreporting in either group; (2) 45% overreporting in both HC users and nonusers; (3) 45% overreporting in HC users only; (4) 80% overreporting in HC users only; (5) 45% overreporting in HC users and 20% in nonusers and (6) 30% overreporting in HC users and 30% underreporting in nonusers. A smoothed surface was fitted to the geometric means of the model simulations using LOESS (*R*^2^=0.9956). (b) Scenario 2: reported condom use from the Partners in Prevention HSV/HIV Transmission Study, HR=1.2. Ninety-one percent of both HC users and nonusers report consistent condom use. Specific misreporting biases are labeled 1 and 2: (1) 30% overreporting in HC users only and (2) 40% overreporting in HC users and 10% in nonusers. Surface fitted using LOESS (*R*^2^=0.9955). c. Scenario 3: 50% condom use, HR=1. Fifty percent of both HC users and nonusers report consistent condom use. Specific points labeled 1–6 refer to the same misreporting behaviors detailed in Fig. 2a and are plotted in Fig. 2d (light gray). Surface fitted using LOESS (*R*^2^=0.9965). (d) Point estimates from Scenarios 1 and 3. Point estimates of HRs observed in the simulated data from Scenarios 1 (dark gray, Fig. 2a) and 3 (light gray, Fig. 2c). Points 1–6 refer to the specific misreporting biases identified in Fig. 2a. Error bars represent 90% of the variation in the model-generated $\hat{\mathrm{HR}}$s at that point, and the dashed black line marks the HR for all HC observed in Heffron et al. (HR=1.98 [1.06–3.68]) [7].

Fig. 2a shows the distribution of observed $\hat{\mathrm{HR}}$s over the complete range of possible condom use misreporting assuming no true association between HC use and HIV acquisition risk (true HR=1.0) and an underlying pattern of reported condom use as described in the Partners HSV/HIV Study data analyzed by Heffron et al. With no misreporting by either HC users or nonusers, or equal misreporting by both, changing the level of condom misreporting does not materially affect the $\hat{\mathrm{HR}}$ ($\hat{\mathrm{HR}}$ close to 1 for *X*–*Y* diagonal, e.g., points 1:$\;\hat{\mathrm{HR}}$=1.0 [0.6–1.5] and 2: $\hat{\mathrm{HR}}$=1.0 [0.7–1.4]). Thus, in this population of serodiscordant couples, misreporting condom use per se is benign to the HC–HIV association if the magnitude of misreporting and direction of misreporting are similar for HC users and nonusers.

If there is a tendency to overreport condom use, but only among HC users, then increases in the apparent excess risk may be observed even without any true biological effect of HC on HIV risk ($\hat{\mathrm{HR}}$>1.0). With 45% overreporting among HC users, the $\hat{\mathrm{HR}}\;$matches that observed by Heffron et al. (point 3: $\hat{\mathrm{HR}}$=2.0 [1.5–2.8]). At higher levels of overreporting, the $\hat{\mathrm{HR}}$ is more inflated — e.g., $\hat{\mathrm{HR}}$=2.8 (2.2–4.0) with 80% overreporting (point 4). However, to reach these levels of spurious results requires that there be completely accurate reporting among the HC nonusers. If HC nonusers also overreport condom use but by less than half as much as the HC group, the $\hat{\mathrm{HR}}$ is still elevated but not to the level observed in Heffron et al. (point 5: $\hat{\mathrm{HR}}$=1.5 [1.0–1.8]) [7].

Apparent $\hat{\mathrm{HR}}$ values more extreme than 2.0 can emerge if HC nonusers tend to underreport condom use. For example, with 30% *under*reporting by nonusers, it is possible to generate a high $\hat{\mathrm{HR}}$ with less overreporting by HC users (point 6: $\hat{\mathrm{HR}}$=1.9 [1.3–2.7]). This artificial doubling in risk can be generated with a wide range of misreporting behaviors by nonusers but always requires a minimum of 20% overreporting by HC users.

### 3.2 Scenario 2: assuming a weak association between HC use and HIV acquisition risk

High observed point estimates are reproducible with less skewed patterns of misreporting when we instead assume a low level of excess HIV risk associated with HC use (HR=1.2; Fig. 2b). The overall pattern of model-estimated $\hat{\mathrm{HR}}$s is the same as the scenario with HR=1.0, but the contours have shifted toward the bottom left quadrant. For example, an $\hat{\mathrm{HR}}$ of 2.0 could be generated by 30% overreporting among HC users only, or 40% overreporting among HC users and 10% among nonusers (Fig. 2b, point 1: $\hat{\mathrm{HR}}$=2.0 [1.5–3.1]; point 2: $\hat{\mathrm{HR}}$=2.0 [1.2–2.8]).

### 3.3 Scenario 3: cohort with 50% condom use

Fig. 2c and d (light gray) shows the distribution of $\hat{\mathrm{HR}}$s across the same parameter space as Fig. 2a and d (dark gray) and with the same true HR=1.0 but with the underlying reported condom use reduced to 50% among both HC users and nonusers. Here, the direction of the relationship between condom misreporting and apparent HIV risk is unchanged, but more extreme misreporting behaviors are required to spuriously generate a large $\hat{\mathrm{HR}}$. At point 3, the $\hat{\mathrm{HR}}\;$is 1.3 (0.9–1.7), much lower than the $\hat{\mathrm{HR}}$=2.0 in the model parameterized with high reported condom use. Even with 80% overreporting by HC users and none by nonusers, the $\hat{\mathrm{HR}}$ is 1.5 (1.1–2.2; point 4). Considerable levels of underreporting by women who do not use HC are required to reproduce $\hat{\mathrm{HR}}$=2.0, e.g., accurate reporting by HC users and 80% underreporting by nonusers, or 80% overreporting by HC users and 40% underreporting by nonusers.

Differential misreporting of coital frequency may also generate elevated risk estimates, but 60% overreporting by nonusers, or a combination of 40% underreporting by HC users and 40% overreporting by nonusers is required to reproduce $\hat{\mathrm{HR}}$=2.0 in the model (see Supplementary Appendix).

## 4 Discussion

Appropriate adjustment for sexual behavior is essential to accurately assess from observational data whether some excess risk of HIV acquisition is attributable to the use of HC. We hypothesized that particular patterns of misreporting sexual behavior could lead to a spuriously high $\hat{\mathrm{HR}}$ even if there is no true association.

We found it possible to observe an artificial doubling in HIV risk — even with no true relationship — through residual confounding due to misreporting. However, this requires substantial and directional misreporting that may not be plausible.

With no overreporting by non-HC users, HC users must overreport condom use by 45% for the observed results to be consistent with no true HC–HIV association. With any overreporting by non-HC users, then even greater misreporting among the HC users would be required to generate $\hat{\mathrm{HR}}$=2.0. This degree of reporting bias, which is highly differential between HC users and nonusers, is not supported by biomarker validation studies which have found that HC users are equally [29], [30] or less [31] likely to overreport condom use as women using nonhormonal methods — contrary to the expected trend. However, model variability is large, and risk estimates within the confidence limits of the primary findings are attainable with less extreme patterns of misreporting.

When HC use is associated with a small increase in HIV acquisition risk, smaller differences in misreporting patterns between the HC users and nonusers are needed to reproduce $\hat{\mathrm{HR}}$=2.0. A small but true HR is only likely to be detectable with statistical significance in a study with a very large sample size but could contribute to biased risk estimates in smaller analyses when combined with substantial misreported condom use.

For many studies of HC and HIV risk, overall condom use in the population was low. In the model scenario with lower overall condom use, with a large degree of misreporting and a strong tendency for HC users to overreport more than the control group, the $\hat{\mathrm{HR}}$ does not reproduce the point estimate observed in the Heffron et al. study, but it can attain the point estimates from some, but not all, other observational studies with medium levels of reported condom use [11], [15], [17], [32]. To artificially generate the higher reported risk estimates, we must assume that non-HC users *under*report their true use of condoms. However, behavioral and epidemiological research indicates that responses tend to overstate condom use and other protective behaviors in questionnaires of sexual behavior [33], [34], and underreporting of condom use seems unlikely in settings where HIV prevention activities emphasize condom use. Although the Heffron et al. analysis has the highest reported condom use of all the observational studies included in recent systematic reviews [3], [4], [5], [6], this is likely to be at least partly related to the study group — serodiscordant couples with mutually disclosed HIV status who had motivation within the partnership to use condoms.

Recent analyses have suggested that DMPA may increase risk for HIV acquisition by 40%–50% [5], [6], [35]. Applying our modeled misreporting scenarios to this risk estimate, differential degrees of condom use misreporting could also produce apparent excess risk (Fig. 2a and c). For example, 20% overreporting by DMPA users and complete accurate reporting by HC nonusers would produce $\hat{\mathrm{HR}}$=1.5. With more modest increases in estimated HIV risk than seen in the Heffron et al. analysis, lower levels of differential reporting accuracy could be sufficient to fully account for the increased risk.

The model makes some key simplifications. Injectable and oral contraceptive use is grouped together, and our model assumes that the amount of misreporting among injectable and oral contraceptive users was the same. Over three quarters of our HC use (78%) represent injectables, and since oral and injectable contraceptives are both considered to be effective methods, social desirability bias that can lead to inaccurate condom use reporting is likely to act to the same degree among oral and injectable users. An additional limitation is that we modeled condom use as a “take” type behavior, where couples use condoms either consistently or never; this approach neglects the partial protection that some women may have received due to mixed condom use.

Sexual behavior data are notoriously difficult to capture accurately yet extremely important for understanding risk levels for HIV acquisition. Our analysis confirms that differences in the amount of misreporting among exposure groups can result in spurious associations but asserts that considerable differences in misreporting would be needed for observed levels of excess risk among HC users to be consistent with no true association between HC use and HIV acquisition risk. Future studies designed to address the question of hormonal contraception and HIV risk must be designed to incorporate multiple methods to assess and validate sexual behavior reports.

## Figures and Tables

**Table 1:** Sexual behavior

Sexual risk group (reported monthly coital frequency)	Proportion of population
0–1	0.221
2–3	0.214
4–6	0.201
7–9	0.119
10–14	0.124
15–30	0.121

Reported monthly coital frequency at each study interval was categorized into six groups to represent the heterogeneity in sexual risk behavior. Each couple is assigned to a category at the start of the simulation, and each month, the coital frequency in the model is randomly selected from within that group.

**Table 2:** Modeling scenarios: HIV risk and contraceptive use

	Scenario 1	Scenario 2	Scenario 3
Underlying HIV risk for HC users			
HR	1.0	1.2	1.0
Contraceptive method			
Injectable hormonal contraception	10.8%	10.8%	10.8%
Oral hormonal contraception	4.0%	4.0%	4.0%
Condoms (consistent use)	91.1%	91.1%	50%
Other	None	None	None

Injectable and oral contraceptive use in the model is assumed to be the same as that reported at enrolment among uninfected women in Heffron et al. (Table 1 in [7]). Condom use in scenarios 1 and 2 is derived from all follow-up intervals in initially uninfected women (Table 2 in [7]); scenario 3 assumes a lower overall level of condom use. Condom use is randomly assigned in the model independently of other contraceptive use. With no misreporting, condoms are assumed to be used consistently by all couples assigned as condom users. Condom misreporting can be varied independently among HC users and nonusers. We assume that there is no contraceptive switching through the simulated follow-up period and no other contraceptive methods in use.

## Appendix A Supplementary data

Supplementary materials Model descriptions and results from sensitivity analyses. Supplementary materials
