# Supplementary material for: Could misreporting of condom use explain the observed association between injectable hormonal contraceptives and HIV acquisition risk?
Source: Contraception. 2017 Apr;95(4):424–30. doi: 10.1016/j.contraception.2016.12.003 (PMC5387890; doi:10.1016/j.contraception.2016.12.003)
Supplement: Supplementary materials — Model descriptions and results from sensitivity analyses. [file mmc1.docx]

**Could misreporting of condom use explain the observed association between injectable hormonal contraceptives and HIV acquisition risk?**

***Supplementary appendix***

*Sensitivity analysis: Misreporting coital frequency*

We repeated the analysis under the null assumption of HR=1.0 to examine the impact of misreporting coital frequency on the observed $\hat{\mathrm{HR}}$.

Misreporting coital frequency is modelled by manipulating the monthly number of sex acts assumed in the model relative to the number reported. This was implemented as $n=\left( 1-\frac{c}{100} \right).n_{m}$, where $n$ is the true monthly coital frequency, $n_{m}$ is the reported coital frequency and $c$ is the misreporting parameter.

Misreporting coital frequency produces the opposite relationship to misreporting condom use (Figure S1). With no misreporting by either HC users or non-users, the $\hat{\mathrm{HR}}$ matches the model input value of HR=1.0 as expected (Figure 2, point 1: $\hat{\mathrm{HR}}$=1.0 [0.6-1.4]). If both HC users and non-users misreport their coital frequency, the observed $\hat{\mathrm{HR}}$ does not substantially deviate from 1 (no effect) while the level of misreporting is the same in both groups (X-Y diagonal, point 2: $\hat{\mathrm{HR}}$=0.9 [0.4-1.6]). The highest $\hat{\mathrm{HR}}$s are observed when there is a high level of over-reporting coital frequency by HC non-users and a high level of under-reporting by HC users (lower right-hand corner, point 3: $\hat{\mathrm{HR}}$=2.1 [1.4-3.4]; point 4: $\hat{\mathrm{HR}}$=2.0 [1.4-3.1]; point 5: $\hat{\mathrm{HR}}$=2.0 [1.1-3.1]). Low $\hat{\mathrm{HR}}$ values are generated by over-reporting among HC users and under-reporting among non-users.

**Figure S1. Model-generated hazard ratios (HRs) with different levels of misreported coital frequency**

Point estimates of HRs observed in the simulated data under different levels of misreporting coital frequency among HC users (15% of women; vertical axis) and non-users (85% of women; horizontal axis). Specific misreporting biases labelled 1-5 are discussed in the text. These are: **(1)** no coital frequency misreporting in either group; **(2)** 45% over-reporting in both HC users and non-users; **(3)** 60% over-reporting in non-HC users only; **(4)** 20% over-reporting in non-HC users and 80% under-reporting in non-users; **(5)** 40% over-reporting in non-HC users and 40% under-reporting in non-users. A smoothed surface was fitted to the geometric means of the model simulations using locally weighted scatterplot smoothing with a quadratic polynomial (LOESS; R^2^=0.9885).
